# Associations between protein intake status and the association of “double burden of malnutrition” in children aged 2–5 years: a health and nutrition survey of selected children in Bengbu City, China

**DOI:** 10.3389/fnut.2025.1564733

**Published:** 2025-07-03

**Authors:** Tongying Zhu, Li Zhang, Rongrong Li, Xiaoqing Li, Hao Zhu, Li Shu

**Affiliations:** School of Public Health, Bengbu Medical University, Bengbu, China

**Keywords:** double burden of malnutrition, obesity/overweight, wasting, stunting, preschool children

## Abstract

**Background:**

Protein intake is widely recognized as a reliable marker for assessing malnutrition. The double burden of malnutrition (DBM) is characterized by the coexistence of malnutrition and overweight, obesity, or diet-related noncommunicable diseases in individuals, families, and populations throughout the life course.

**Methods:**

Children were categorized into quartiles based on the protein index. World Health Organization (WHO) Anthro software was used to assess child growth and development, enabling the prediction of DBM prevalence. Nutrient intake, the effect on DBM were assessed using both a multifactorial logistic regression model and a binary logistic regression correction model.

**Results:**

The prevalence of DBM increased with higher protein index quartiles. In the regression model, the risk of DBM was significantly higher in the second (Q_2_) and fourth (Q_4_) quartiles for both boys (Q_2_: 1.934, 95% CI: 1.210–3.091; Q_4_: 1.653, 95% CI: 1.016–2.687) as well as in the girls (Q_2_: 1.963, 95% CI: 1.263–3.052; Q_4_: 1.366, 95% CI: 0.857–2.178). In the multivariate models, the association between the protein index and DBM risk persisted as significant even after adjusting for confounding factors.

**Conclusion:**

This study revealed a strong correlation between the protein index and DBM prevalence in preschool children aged 2–5 years.

## 1 Introduction

In recent years, global demographic shifts, economic growth, rising incomes, and urbanization have substantially influenced nutritional status and have led to significant changes in both the quality and quantity of human diets as well as nutrition-related epidemiology worldwide (1, 2). As stated by the United Nations Children’s Fund, an estimated 6.7% (45.4 million) children below the age of 5 years experienced wasting in 2020, whereas 22% (149.2 million) were stunted (3). Childhood stunting is linked to various short-and long-term adverse outcomes, including increased childhood morbidity and mortality, impaired cognitive development, increased risks of obstetric complications and maternal mortality among women of childbearing age, diminished productivity and earnings in adulthood, and intergenerational effects on health and nutrition (4). To address the double burden of nutrition-related health issues among school-aged children, the World Health Organization (WHO) launched the Nutrition-Friendly School Initiative (NFSI) in 2006 (5). However, the classification of undernourished children varies across studies due to differences in the indicators and cutoff points used. The indicators of child undernutrition employed in the current double burden of malnutrition (DBM) research include the weight-for-age Z-score (WAZ), height-for-age Z-score (HAZ), BMI-for-age Z-score (BAZ), simple BMI, or combinations of WAZ, HAZ, and weight-for-height Z-score (WHZ) (6, 7). By identifying potential factors that influence growth and development, employing early monitoring of growth and development indicators, improving the structure and quality of children’s diets, and fostering a positive and healthy dietary environment, the incidence of overweight/obesity and cardiovascular disease in adulthood can be reduce (8).

Proteins are a critical nutrient for the growth and development of preschool-aged children, and inadequate protein intake can lead to protein-energy malnutrition, anemia, and immune dysfunction (9). Proteins are essential for various physiological processes, the human body requires adequate proteins, whether derived from plant or animal sources, to support growth and development. For absorption and utilization, proteins must first be digested and broken down into amino acids (10). Of >20 amino acids, 8 are categorized as “essential amino acids” because the human body cannot synthesize them in adequate quantities and must obtain them from external food sources (11). Plasma levels of essential amino acids (12) are lower in children with developmental delays. Furthermore, protein intake is strongly linked to weight-related parameters, including the WAZ, WLZ, and BAZ, even after adjustment for potential confounders.

Research has demonstrated that different sources of protein, such as dairy and nondairy proteins, have varying effects on preschool children, with dairy proteins being primarily associated with weight gain (13). This finding highlights the close relationship among dietary proteins and the growth and development of children. Additionally, some studies have suggested that consuming protein in accordance with the recommended intake may extend the average lifespan by 1.19 years or approximately 1.8% (14). Given the widespread prevalence of malnutrition and its considerable associated health and economic costs, DBM continues to be a significant public health challenge, especially in low-and middle-income countries, where the prevalence of overweight and obesity has risen over the past two decades (15). Relative to high-income countries the incidence of DBM in children has increased by 30% in many developing countries (16). Despite this trend, research on the relationship between dietary protein intake and DBM is lacking, both domestically and internationally. Therefore, greater attention must be paid to this phenomenon, with a focus on investigating the factors contributing to the simultaneous occurrence of stunting and overweight. This research is essential for providing a robust theoretical foundation for formulating policies aimed at promoting the healthy development of children. In this study, dietary protein intake was used as an entry point to study the relationship between, along with development in DBM. It evaluates the development and maturation of children through DBM, and studies the key influencing factors of stunting, wasting and overweight in children, so as to provide some data support for the promotion of children’s healthy growth.

## 2 Materials and methods

### 2.1 Study population

**Figure 1:**
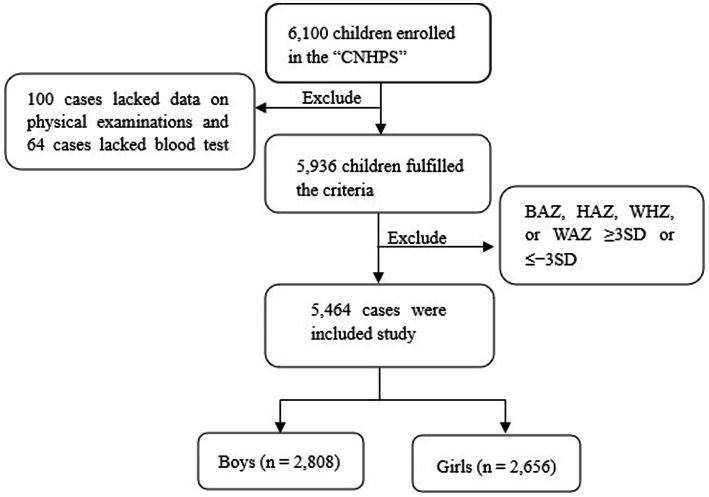
Flowchart of participant selection for this study.

This cohort study was conducted as a part of the “Childhood Nutrition and Health Promotion Study” (CNHPS) in Bengbu City. The following criteria were used for inclusion: (1) Voluntary participation in the study; (2) healthy preschool children aged 2–5 years; (3) absence of prosthetic devices, surgical staples, or high-density materials in the examined areas; and (4) presence of complete survey data. The exclusion criteria were as follows: (1) Refusal to voluntarily cooperate in the medical examination; (2) joint injuries or skin lacerations; and (3) congenital diseases, hereditary diseases, chronic metabolic diseases, or psychological disorders. (4) Some children did not participate in the survey because of illness, absence from school, etc. After screening and organizing the data, a total of 187 children were missing data. A total of 5,464 children (2,808 boys and 2,656 girls) aged 2–5 years incorporated in this study are shown in (Figure 1). Initially, 6,300 health questionnaires for preschool children were distributed, with 6,100 questionnaires collected, yielding a response rate of 96.82%. Of these, 5,936 children underwent on-site physical examinations and bone density tests, with an effective participation rate of 97.31%. Among them, 164 cases were excluded due to missing data (100 with incomplete physical examination data and 64 with missing blood test results). Additionally, 472 outliers were excluded from the analysis (those with BAZ, HAZ, WHZ, or WAZ ≥ 3 SD or <−3 SD). Following these exclusions, 5,464 children aged 2–5 years were selected for the final analysis. This cohort comprised 2,808 boys and 2,656 girls, with 24 children aged 2 years, 134 children aged 3 years, 2,173 children aged 4 years, and 3,133 children aged 5 years. The study protocol followed t the standards established in the Declaration of Helsinki and secured approval from the Ethics Committee at Bengbu Medical University. (Ethics Committee No. [2023] 261).

### 2.2 Questionnaire survey and dietary information survey

Data collection occurred via in-person interviews conducted by trained personnel. The self-designed Preschool children’s Health Questionnaire was used to conduct a survey among kindergartens in Bengbu City, and the data collected from preschool children were subsequently summarized and analyzed. The questionnaire collected the following data: (1) General demographic details, such as the child’s gender age, grade, place of residence, parents’ height, parental education parents’ job status, and monthly family earnings., whether the child is an only child, whether the child is a left-behind child, and the identity of the current caregiver. (2) The 3-day 24-h dietary recalls were conducted non-consecutively, spanning two weekdays (Monday–Friday) and one weekend day (Saturday/Sunday). Tools such as dietary atlases, the plate model, the food photo collection, nutritional-component display cards, and food pyramid models were employed to assist caregivers in accurately recalling the types and quantities of the children’s food.

Dietary preferences were evaluated using the 24-h recall method, where the variety of foods consumed by, a child in the past 24-h was recorded. During school hours, researchers collected meal recipes from kindergartens and recorded the children’s breakfast and lunch based on the kindergarten’s menu. For meals consumed at home, precoded questionnaires were distributed to the primary caregivers, who provided information on the children’s food intake, including the food frequency, timing, types, and portion sizes. The survey data were analyzed using Nutrition Calculator software 2.7.3.

### 2.3 Anthropometric measurements and laboratory examination

Trained investigators recorded the height (cm) along with the weight (kg) of the children by employing an automated height and weight measuring device (Hendin Technologies DMH-301). Participants were asked to take off their shoes and jackets and to wear comfortable, loose-fitting garments to obtain accurate measurements. Waist Circumference (WC) was measured using a soft tape placed around the navel, with the assessment conducted after exhalation. WC was recorded in centimetres, and all data were recorded to two decimal places for precision.

Early in the morning, professionals collected fasting blood samples from the participants (after at least 8 h of fasting) to measure hemoglobin levels by using a fully automated haemoglobin analyzer (BeneCheck Hemoglobin Detector, Zhengzhou Aosikang Electronic Technology Co., Ltd., model PD-G017). Calcium, iron, and zinc intake was assessed on the basis of dietary surveys.

### 2.4 Definitions

The WHO defines DBM as “the coexistence of malnutrition and overweight, obesity, or diet-related noncommunicable diseases in individuals, families, and populations throughout the life course.” In this context, malnutrition includes wasting, stunting, and micronutrient deficiencies (5). Previous studies have defined DBM by using only anthropometric data, such as stunting and overweight (17), or in combination with the hemoglobin concentration (18, 19) and, less frequently, with biomarkers of micronutrient deficiency (20, 21). Although no universal consensus on the definition of DBM is available, the WHO criteria are widely accepted, both nationally and internationally, as reference standards. This study also adopted the WHO criteria for defining DBM. In this study, stunting, wasting, micronutrient deficiencies, iron deficiency anemia, and overweight/obesity were selected as indicators of DBM (22–24), enabling a comprehensive and integrated assessment of the prevalence of DBM and the factors influencing it.

The official WHO software programs WHO Anthro and WHO Anthro Plus v 1.0.4 (Department of Nutrition World Health Organization, Geneva, Switzerland) were applied to evaluate the WHZ, HAZ, WAZ, and BAZ for each participant. The nutritional situation of the children was conducted based on these four Z-scores according to the 2007 WHO recommended guidelines for the progress and evolution of preschool children (5). Specifically, a WHZ (or BAZ) of <−2 was used to identify wasting, a HAZ of <−2 was used to indicate stunted growth and a WAZ score of <−2 was used to denote low body weight. Conversely, a BAZ score of >2 was used to denote overweight.

According to the diagnostic criteria for anemia in preschool children (25), in the present study, anemia was defined as follows: for children aged 6 to 59 months, hemoglobin levels less than 110 g/L are considered low, while levels below 115 g/L apply to children aged 5 to 11 years. Hemoglobin levels below these thresholds are considered to indicate deficiency.

As outlined in the 2016 version of the Dietary Nutrient Reference Intake for Chinese Residents (26), the average protein requirement for boys and girls aged 2–5 years is between 25 and 30 g/d. Intake below this standard is considered deficient. The protein index represents total daily protein intake (g/day) adjusted for age-and sex-specific energy requirements. It was calculated as: Protein Index = Observed Protein Intake (g/day) /Recommended Protein Intake for Age-Sex Group (g/day) (27).

Left-behind children refer to minor children who have been separated from one or both parents for at least 6 months due to their parents working away from home (28).

### 2.5 Statistical analyses

General demographic data and information on children’s eating behavior were entered using Epidata 3.1 software. Data analysis was performed using SPSS 25.0 software (IBM SPSS Inc., Chicago, IL, USA), MedCalc Version20 (DEMO) software (MedCalc Software Ltd., Ostend, Belgium), and children’s food and nutritional intake was calculated using Nutritional Calculator V1.6 software. Z-scores for children’s growth and development were calculated using WHO Anthro and WHO 14 Anthro Plus software. GraphPad Prism 9.0 for Windows (GraphPad Software, San Diego, CA, USA) was used to generate histograms of the prevalence of protein index quartiles for boys and girls, multifactorial line graphs depicting the occurrence of DBM in boys and girls aged 2 to 5 years, and forest plots for logistic regression analyses in boys and girls. ROC curve analysis was conducted to assess the sensitivity, specificity, and Youden index for each indicator’s ability to differentiate DBM. The area under the curve (AUC) was compared to evaluate the predictive performance of various indicators related to DBM risk. A Z test was employed to analyse the AUC, with *p*-values < 0.05 indicating statistical significance. The Kolmogorov–Smirnov test was utilized to analyse data normality, and the chi-square test was employed to analyse categorical data. Measures assumed to have a normal distribution are illustrated as ( $\bar{x}$ ± s). An independent t-test was conducted to compare nutrient intake values between boys and girls. Measures with skewed distribution are presented as *M* (P_25_, P_75_), and differences in physical development between anemic and nonanemic groups were analyzed using the Mann–Whitney U test. Categorical variables are expressed as percentages and frequencies, and the chi-square test was examined to evaluate group differences. Protein indices were categorized into four quartiles (Q_1_, Q_2_, Q_3_, and Q_4_) for data analysis. Multifactorial logistic regression was applied to explore the correlation between DBM risk and protein index across the quartile groups. A series of binary logistic regression models were employed to adjust for potential confounders: Model 1 adjusted for ethnicity, left-behind status, living location, and anemia; Model 2 further adjusted for height and weight Model 1 served as the foundation; Model 3 further adjusted for carbohydrates, fat, and energy intake based on Model 2.

## 3 Results

### 3.1 Basic characteristics of the study population in our study

Table 1 presents the demographic information. Among the participants, the prevalence of DBM was 0.61% at age 2, 3.94% at age 3, 38.48% at age 4, and 56.97% at age 5. DBM was 746 boys (26.57%) and 720 girls (27.11%). Significant differences were observed in anemic, Father’s education, wasting, stunting, and overweight/obesity between the DBM and non-DBM groups (*p* < 0.05).

**Table 1:** Basic characteristics of the study population in our study [*n* (%)].

Characteristics	DBM (*n* = 330)	Non-DBM (*n* = 5,134)	*χ* ^2^	*p*
Age, *n* (%)			3.476	0.176
2	2 (0.61)	22 (0.43)		
3	13 (3.94)	121 (2.36)		
4	127 (38.48)	2046 (39.85)		
5	188 (56.97)	2,945 (57.36)		
Gender			1.187	0.276
Boys	160 (48.48)	2,648 (51.58)		
Girls	170 (51.52)	2,486 (48.42)		
Ethnicity			0. 728	0.394
Han Chinese	326 (98.79)	5,094 (99.22)		
Minority	4 (1.21)	40 (0.78)		
Residence, *n* (%)			0.221	0.638
Rural	74 (22.42)	1,095 (21.33)		
Urban	256 (77.58)	4,039 (78.67)		
Left-behind children, *n* (%)			0.228	0.663
Yes	76 (23.03)	1,242 (24.19)		
No	254 (76.97)	3,892 (75.81)		
Anemic			78.634	0.000
Yes	138 (41.82)	218 (4.25)		
No	192 (58.18)	4,916 (95.75)		
Bone density status			2.850	0.240
Low bone density	3 (1.08)	78 (1.50)		
Normal bone density	23 (8.30)	308 (5.94)		
Adequate bone density	251 (90.61)	4,801 (92.56)		
Educational level of caregivers			0.578	0.749
Junior high school and below	103 (31.21)	1,556 (30.31)		
High school/secondary school	144 (43.64)	2,349 (45.75)		
College and above	83 (25.15)	1,229 (23.94)		
Caregiver occupations			2.389	0.303
Commercial agroforestry personnel	220 (66.67)	3,301 (64.30)		
National staff	3 (0.91)	104 (2.03)		
Others	107 (32.42)	1729 (33.68)		
Father’s education			6.490	0.039
Junior high school and below	87 (26.36)	1,208 (23.53)		
High school/secondary school	94 (28.48)	1816 (35.37)		
College and above	149 (45.15)	1,208 (23.53)		
Mother’s education			1.903	0.386
Junior high school and below	89 (26.97)	1,214 (23.65)		
High school/secondary school	119 (36.06)	1918 (37.36)		
College and above	122 (36.97)	2002 (38.99)		
Monthly household income/(yuan)			0.157	0.901
<10,000	219 (66.36)	3,390 (66.03)		
>10,000	111 (33.64)	1744 (33.97)		
Wasting			311.839	<0.001
Yes	100 (30.30)	268 (5.22)		
No	230 (69.70)	4,867 (94.78)		
Stunting			756.879	<0.001
Yes	158 (47.88)	284 (5.53)		
No	172 (52.12)	4,850 (94.47)		
Overweight/Obesity			262.049	<0.001
Yes	142 (43.03)	597 (11.59)		
No	188 (56.97)	4,537 (88.41)		

### 3.2 Demographics of the study population by nutrient intake and protein index quartiles

**Figure 2:**
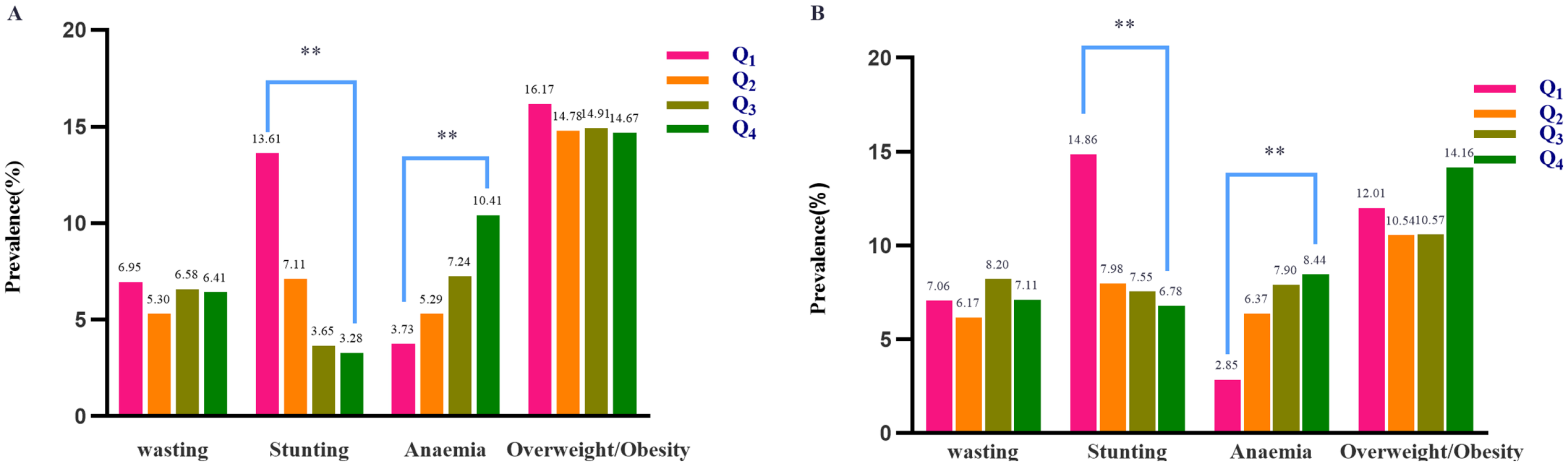
Prevalence of wasting, stunting, anaemia, and overweight/obesity according to quartiles of protein intake among **(A)** boys and **(B)** girls. Statistically significant differences between groups are indicated as follows: *p < 0.05, **p < 0.01.

Table 2 Carbohydrate intake was significantly higher in children aged 3 years than in those of other ages. There may be differences in the accuracy of self-reporting of food intake by children of different ages or reporting on their behalf by parents. For example, the diet of 3-year-old children often relies more on the arrangements and descriptions of their parents. Parents may find it easier to observe and record their children’s intake of carbohydrate-rich foods (such as staple foods), but they may not accurately estimate the specific intake of protein, fat, and other nutrients. As children get older, they have more opportunities for independent eating and a wider variety of food choices. Parents may find it difficult to accurately track all food intake, leading to changes in the accuracy of reporting, and thus the data presents such a trend. By contrast, fat, protein, energy, calcium, iron, and zinc intake consistently increased with age. The incidence of wasting, stunting, anemia, and overweight/obesity was assessed across quartiles of protein intake. Statistically notable differences were found among the protein quartile groups in anemia and stunting, except for the incidence of wasting, and overweight/obesity, which did not significantly vary (Figure 2).

**Table 2:** Estimated daily nutrient intake for boys and girls aged 2–5 years in the “Childhood Nutrition and Health Promotion Study” (CNHPS) (*N* = 5,464) (M [P_25_, P_75_]).

Nutrient intake	2 years (*n* = 24)	3 years (*n* = 134)	4 years (*n* = 2,173)	5 years (*n* = 3,133)
Carbohydrate, g/d	165.56 (113.07, 216.87)	179.66 (115.42, 249.85)	179 (120.10, 229.43)	179 (120.10, 229.43)
Fat, g/d	33.35 (29.30, 36.05)	43.15 (34.10, 52.50)	52.10 (45.30, 55.70)	52.10 (45.30, 55.70)
Protein, g/d	21.15 (17.85, 24.30)	29.30 (22.60, 38)	37.90 (32.20, 41.40)	37.90 (32.20, 41.40)
Energy, kcal/d	1,144 (897.05, 1380.50)	1324.75 (1023.30, 1614.50)	1,406 (1151.90, 1673.30)	1,406 (1151.90, 1673.30)
Calcium, mg/d	81 (76.50, 89.50)	102.50 (88, 158)	170 (124, 202)	170 (124, 202)
Iron, mg/d	0.98 (0.85, 1.12)	1.34 (1.12, 2.53)	2.94 (1.82, 3.80)	2.94 (1.82, 3.80)
Zinc, mg/d	3.22 (2.35, 3.60)	4.75 (3.60, 6.70)	7.20 (5.50, 8.30)	7.20 (5.50, 8.30)

The demographics of the study group across the quartiles of the protein index are presented in Table 3 marked differences (*p* < 0.05) were observed in several indicators, including stunting, HC, WC, BMI levels, carbohydrate intake, energy intake, iron intake, and zinc intake, among the groups. Furthermore, the prevalence of anemia, body weight, fat intake, and calcium intake exhibited an increasing trend with higher protein index quartiles (all *p* < 0.05).

**Table 3:** Demographic characteristics of children aged 2–5 years according to quintiles of protein intake in the CNHPS cohort, *n* (%).

Variables	Quintiles of protein intake (g/d) (boys)				*p*	Quintiles of protein intake (g/d) (girls)				*p*
	Q_1_ (<37.80)	Q_2_ (37.80–44.90)	Q_3_ (44.90–51.58)	Q_4_ (>51.58)		Q_1_ (<38.00)	Q_2_ (38.00–45.00)	Q_3_ (45.00–52.08)	Q_4_ (>52.08)	
*N*	705	717	684	702		666	664	662	664	
Age, *n* (%)					<0.001					<0.001
2	17 (2.41)	9 (1.31)	13 (1.91)	20 (2.85)		7 (1.05)	8 (1.20)	15 (2.27)	18 (2.71)	
3	53 (7.52)	16 (2.23)	30 (4.48)	44 (6.27)		47 (7.06)	10 (1.51)	85 (12.84)	16 (2.41)	
4	555 (78.72)	400 (56.06)	171 (25.00)	58 (8.36)		546 (81.98)	340 (51.21)	154 (23.26)	30 (4.52)	
5	80 (11.35)	290 (40.40)	470 (68.71)	580 (82.52)		66 (9.91)	306 (46.08)	408 (61.63)	600 (90.36)	
Residence, *n* (%)					0.785					0.550
Rural	543 (77.02)	564 (78.66)	528 (77.19)	553 (77.77)		537 (80.63)	520 (78.31)	517 (78.10)	533 (80.27)	
Urban	162 (22.98)	153 (21.34)	156 (22.81)	149 (22.23)		129 (19.37)	144 (21.69)	145 (21.90)	131 (19.73)	
left-behind children, *n* (%)					0.429					0.645
Yes	532 (75.46)	541 (75.45)	535 (78.22)	524 (74.64)		513 (77.03)	498 (75.00)	508 (76.74)	495 (74.55)	
No	173 (24.54)	176 (24.55)	149 (21.78)	178 (25.36)		153 (23.97)	166 (25.00)	154 (23.26)	169 (25.45)	
Wasting, *n* (%)	49 (6.95)	38 (5.30)	45 (6.58)	45 (6.41)	0.735	47 (7.06)	41 (6.17)	55 (8.20)	48 (7.11)	0.578
Stunting, *n* (%)	96 (13.61)	51 (7.11)	25 (3.65)	23 (3.28)	<0.001	99 (14.86)	53 (7.98)	50 (7.55)	45 (6.78)	<0.001
Anemia, *n* (%)	27 (3.73)	37 (5.29)	51 (7.24)	71 (10.41)	<0.001	19 (2.85)	41 (6.37)	53 (7.90)	57 (8.44)	<0.001
Overweight/ Obesity, *n*(%)	114 (16.17)	106 (14.78)	102 (14.91)	103 (14.67)	0.904	80 (12.01)	70 (10.54)	70 (10.57)	94 (14.16)	0.196
HC, (cm)	50 (49, 51)	50 (49.20, 51.50)	51 (49.50, 52.50)	50 (49.30, 51.50)	<0.001	50 (49, 51.)	50 (49.00, 51.50)	51 (49.50, 52.00)	50 (49.20, 51.30)	<0.001
Weight, (kg)	16 (14.70, 17.30)	17.20 (15.70, 19.00)	19 (17.00, 20.50)	20 (19.00, 21.50)	<0.001	15.30 (14.00, 16.80)	17 (15.10, 19.00)	18 (16.90, 20.00)	20 (18.20, 21.50)	<0.001
Height, (cm)	100 (97.00, 103.50)	104.90 (100.50, 109.60)	110 (106.00, 113.00)	113.50 (110.50, 116.50)	0.906	99 (95.40, 102.50)	104.90 (100, 109)	108.50 (105, 112)	113 (110, 116)	0.894
BMI (kg/m2), M(P_25_, P_75_)	15.91 (1, 510, 16.88)	15.79 (14.89, 16.74)	15.63 (14.61, 16.57)	15.67 (14.72, 16, 64)	<0.001	15.74 (14.82, 16.76)	15.56 (14.60, 16.41)	15.38 (14.46, 16.57)	15.50 (14.52, 16.63)	<0.001
Carbohydrate (g/d), M(P_25_, P_75_)	129.75 (111.10, 179.96)	206.70 (112.90, 267.17)	208.35 (172.30, 236.07)	175.02 (118.25, 213.20)	<0.001	129.75 (107.90, 177.96)	202 (112.78, 259.60)	207.67 (162.60, 238.06)	179.90 (125.32, 216.00)	<0.001
Fat (g/d), M(P_25_, P_75_)	45.30 (39.80, 48.90)	55.70 (54.60, 57.00)	61.10 (59.80, 64.60)	70.80 (68.70, 73.20)	<0.001	44.50 (38.30, 48.90)	55.80 (54.60, 57.00)	62.10 (59.97, 64.60)	70.80 (68.80, 73.20)	<0.001
Energy (g/d), M(P_25_, P_75_)	1169.60 (1035.10, 1364.10)	1546.00 (1235.60, 1814)	1, 671 (1502.00, 1808.05)	1, 659 (1398.60, 1855.40)	<0.001	1167.30 (995.82, 1347.60)	1525.10 (1233.85, 1782.40)	1665.40 (1462.70, 1815)	1680.60 (1432.40, 1871.65)	
Calcium (mg/ d), M(P_25_, P_75_)	171 (154, 186)	153 (123, 336)	249 (231, 277)	501 (440, 576)	<0.001	170 (95, 186)	212 (124, 337)	252 (234, 279)	495 (431, 564)	<0.001
Fe (mg/d), M(P_25_, P_75_)	2.94 (2.47, 3.17)	2.39 (1.80, 10.35)	6.67 (5.62, 8.06)	17.05 (14.52, 25.08)	<0.001	2.94 (1.19, 3.17)	4.69 (1.82, 10.53)	6.85 (5.64, 8.10)	17.03 (14.12, 23.98)	<0.001
Zinc (mg/d), M(P_25_, P_75_)	7.30 (6.40, 7.80)	6.40 (5.40, 10.90)	9.50 (8.80, 10.00)	13.20 (12.40, 14.20)	<0.001	7.20(4.10, 7.80)	8.40(5.50, 10.90)	9.50(8.90, 10.10)	12.30(13.10, 13.90)	<0.001

### 3.3 Line analysis of the incidence of DBM

**Figure 3:**
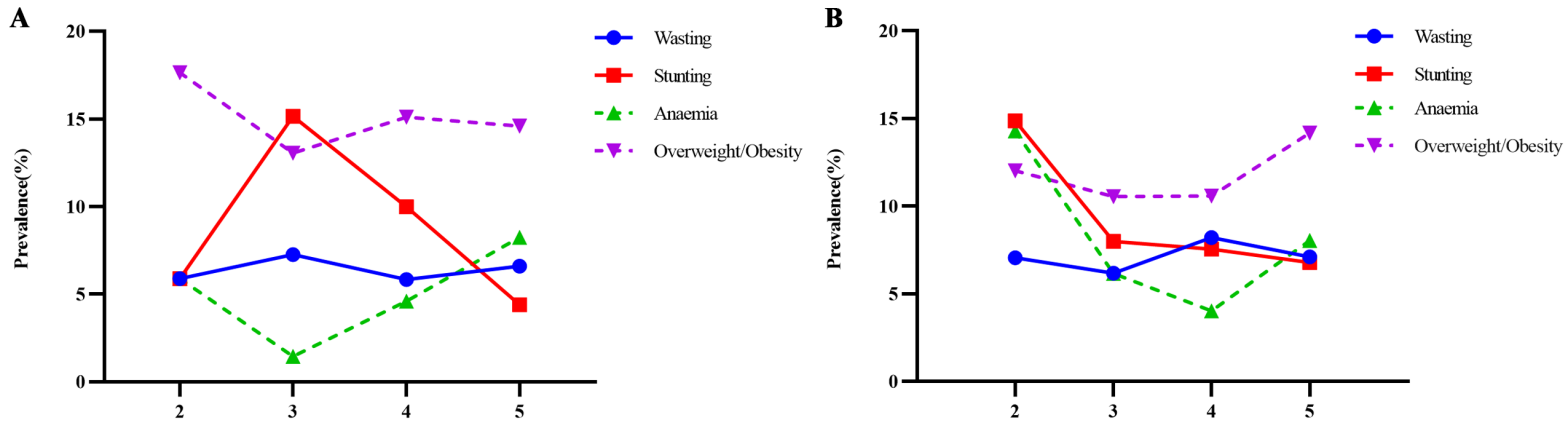
**(A)** boys and **(B)** girls show the prevalence of DBM among boys and girls aged 2-5 years.

Figure 3 presents the trends of the occurrence of DBM among preschool children. In the boys, the incidence of wasting was the lowest (5.83%) at 4 years of age, whereas the occurrence of stunting was the highest at (15.14%) at 3 years of age. The anemia was the highest (8.24%) at 5 years of age, with a marked decrease to (1.45%) at 3 years of age. Overweight/obesity increased (15.99%) at 2–4 years of age and decreased (14.59%) at 5 years of age. In the girls, the rate of wasting was the lowest (6.82%) at 4 years of age and the highest (15.38%) at 3 years of age. Stunting was the lowest (5.77%) at 5 years of age. The prevalence of anemia was the highest (14.28%) at 2 years of age and the lowest (4.03%) at 4 years of age. The disease rate of overweight/obesity was the highest (12.35%) at 5 years of age. Girls exhibited a higher prevalence of stunting than boys in the 2, 4, and 5 years age groups. However, the boys in the all-year age group exhibited a significantly higher prevalence of overweight/obesity than the girls.

### 3.4 Logistic regression analysis of risk factors for DBM

**Figure 4:**
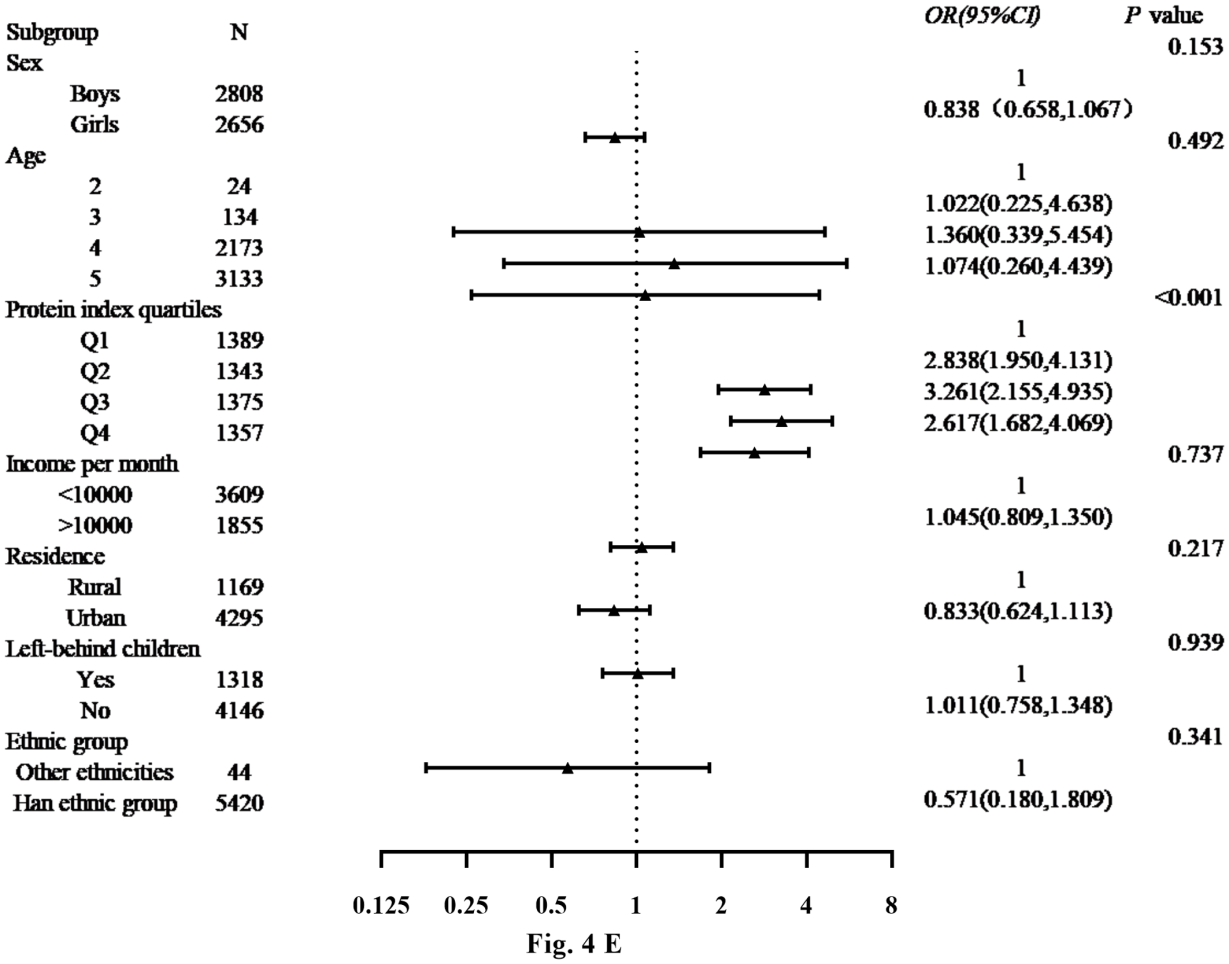
E Binary logistic regression analysis of DBM.

Figure 4 displays the findings from the binary logistic regression analysis of risk factors for DBM in the boys and girls. The analysis revealed significant associations between protein index quartiles for boys and girls. For the girls, the protein index was significantly associated with DBM in the quartilesQ_2_ (OR: 2.838, 95% CI: 1.950–4.131), Q_3_ (OR: 3.261, 95% CI: 2.155–4.935) and Q_4_ (OR: 2.617, 95% CI: 1.682–4.069) compared with the quartile Q_1_. Among the other variables, being left-behind child and family income per month were identified as a contributing factor to DBM (OR: 1.011, 95% CI: 0.758–1.348; OR: 1.045, 95% CI: 0.809–1.350). In addition, place of residence and ethnicity are considered risk factors for DBM (OR: 0.833, 95% CI: 0.624–1.113; OR: 0.571, 95% CI: 0.180–1.809).

### 3.5 Connection between protein index quartiles and the prevalence of DBM

Table 4 shows the association between protein index quartiles and the risk of DBM in the boys and girls. Within this regression model, when the Q_1_ quartile of the protein index was used as the reference group (OR = 1), the risk of DBM was notably greater in the Q_2_ and Q_4_ quartiles groups in the boys (Q_2_: 1.934, 95% CI: 1.210–3.091; Q_4_: 1.653, 95% CI: 1.016–2.687) as well as in the girls (Q_2_: 1.963, 95% CI: 1.263–3.052; Q_4_: 1.366, 95% CI: 0.857–2.178). In the multivariate models, the relationship between the protein index and the risk of DBM continued to be significant even after adjusting for potential confounders.

**Table 4:** Risk of DBM across protein index quartiles.

Variables	Quintiles of protein intake (g/d) (boys)				*p*	Quintiles of protein intake (g/d) (girls)				*p*
	Q_1_ (<37.80)	Q_2_ (37.80–44.90)	Q_3_ (44.90–51.58)	Q_4_ (>51.58)		Q_1_ (<38.00)	Q_2_ (38.00–45.00)	Q_3_ (45.00–52. 08)	Q_4_ (>52.08)	
(*n*, %)	28 (17.50)	54 (33.75)	34 (21.25)	44 (27.50)		32 (18.82)	62 (36.47)	31 (18.24)	45 (26.47)	
Unadjusted	1.00(ref)	1.934 (1.210–3.091)	1.216 (0. 729–2.028)	1.653 (1.016–2.687)	<0.001	1.00(ref)	1.963 (1.263–3.052)	0.926 (0.558–1.537)	1.366 (0.857–2.178)	<0.001
Model 1	1.00(ref)	1.482 (0.989–2.734)	1.054 (0. 605–1.834)	1.199 (0. 703–2.042)	<0.001	1.00(ref)	1.648 (1.159–2.747)	0. 817 (0.474–1.408)	1.242 (0.749–1.988)	<0.001
Model 2	1.00(ref)	1.247 (0.709–2.193)	1.057 (0. 713–1.835)	1.211 (0.878–2.349)	<0.001	1.00(ref)	1.561 (0.925–2.635)	0.899 (0.567–1.660)	1.227 (0.709–1.973)	<0.001
Model 3	1.00(ref)	1.246 (0.707–2.190)	1.065 (0.825–1.959)	1.210 (0.838–2.347)	<0.001	1.00(ref)	1.383 (0.733–2.608)	0.748 (0.388–1.441)	1.225 (0.706–1.971)	<0.001

Quartile 1 (Q_1_) was used as the reference category for both boys and girls.

Model 1: Adjusted for years, ethnicity, and place of residence.

Model 2: Adjusted for height and weight based on Model 1.

Model 3: Adjusted for wasting, stunting, overweight/obesity, anemia based on Model 2.

**Figure 5:**
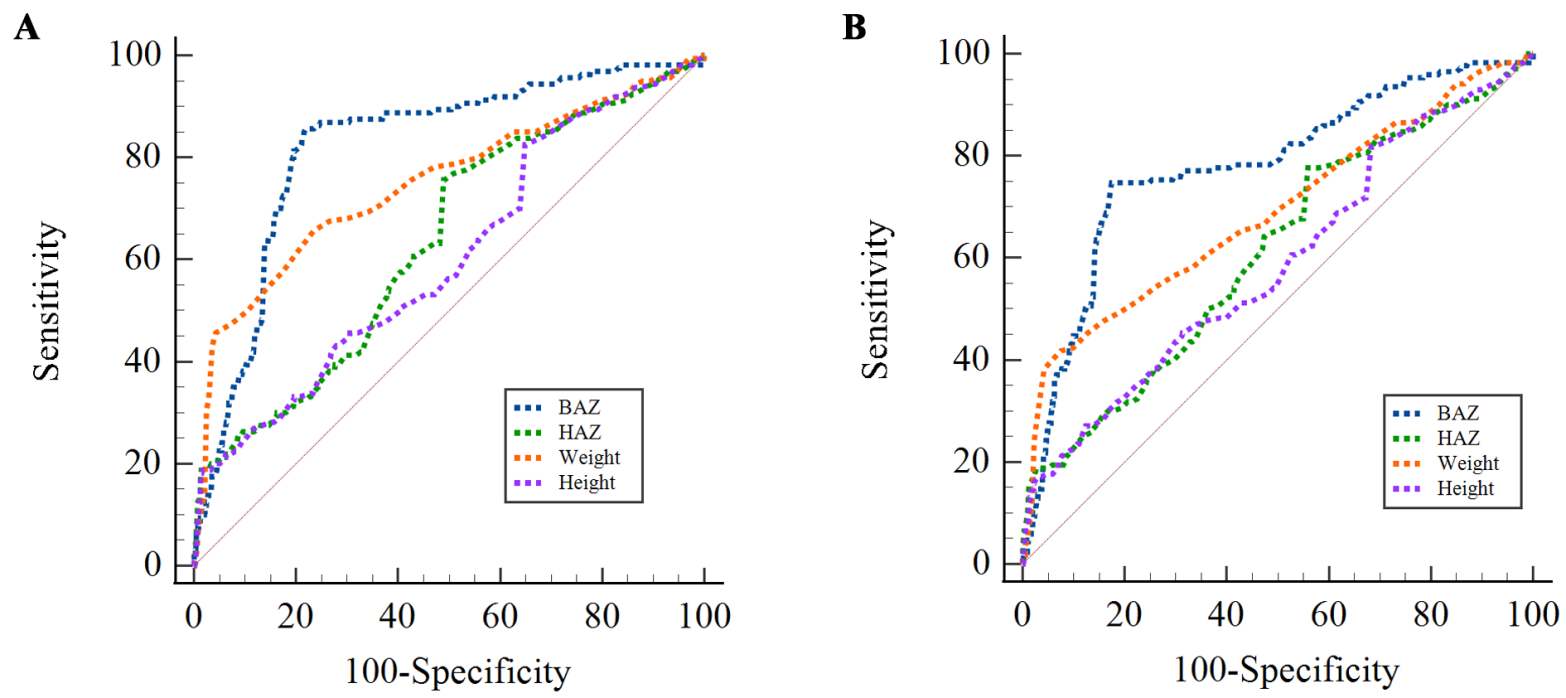
ROC curve of DBM in boys and girls. **(A)** Boys. **(B)** Girls.

Table 5 and Figure 5 show the findings from the ROC curve analysis of DBM including the cut-off values, sensitivity, specificity, Youden index, and AUC values. The AUC for all these indices exceeded 60%, indicating their diagnostic significance for DBM. The cut-off value for height was 110.40 for boys and 97.00 for girls. The BAZ of boys demonstrated the highest AUC value (0.822, 95% CI: 0.807 to 0.836), followed by girls (0.778, 95% CI: 0.762 to 0.794). In comparison, the AUC for height in girls (0.618, 95% CI: 0.600 to 0.635) was greater than that for boys (0.603, 95% CI: 0.584 to 0.621). The ROC curve analysis demonstrated that the protein index (AUC = 0.655, 95% CI: 0.643–0.668) exhibited significantly superior predictive performance for DBM compared to single nutritional indicators (e.g., HAZ, AUC = 0.635). In the discussion, we supplemented the screening strategy for high-risk populations: children with protein intake exceeding 50 g/day should be prioritized for comprehensive DBM assessment. For future research, integrating the protein index with gut microbiome biomarkers, inflammatory markers, and other parameters (29) could further improve the AUC value.

**Table 5:** ROC curve analysis of DBM in boys and girls.

Variables	Cut-off value	Sensitivity (%)	Specificity (%)	Youden index	AUC(95%CI)	*Z*	*p*
Boys							
Height	110.40	82.50	76.16	0.177	0.603 (0.584–0.621)	4.281	<0.001
Weight	16.10	65.62	76.55	0.422	0.749 (0.733–0.765)	10.467	<0.001
HAZ	0.12	75.62	51.10	0.267	0.635 (0.617–0.653)	5.940	<0.001
BAZ	0.98	85.62	78.10	0.637	0.822 (0.807–0.836)	18.733	<0.001
Girls							
Height	97.00	67.06	87.69	0.148	0.618 (0.600–0.635)	3.616	<0.001
Weight	15.10	61.76	92.60	0.344	0.689 (0.671–0.706)	7.801	<0.001
HAZ	0.23	77.65	84.13	0.218	0.610 (0.591–0.629)	4.746	<0.001
BAZ	0.98	74.71	82.58	0.573	0.778 (0.762–0.794)	5.909	<0.001

## 4 Discussion

The results from population-based cohort research have indicated that the risk of DBM rises with protein index quartiles (10, 13, 30). After adjustment for confounders, the association between DBM risk and the protein index remained significant. DBM is a unique global challenge, highlighting the need for interventions that address both the rising rates of obesity in urban areas and the persistent problem of undernutrition in rural China. Between 1985 and 2014, the prevalence of stunting gradually decreased from 16.4 to 2.3%, while the prevalence of thinness reduced from 8.4 to 4.0%. Conversely, the prevalence of overweight substantially increased from 1.1 to 20.4% (31). The evolution of childhood undernutrition and overnutrition in China can be categorized into three phases: First, from 1985 to 1995, malnutrition, which is characterized by stunting and wasting, was the main public health issue in China. Although the occurrence of stunting was rapidly declining, overweight was relatively uncommon and increased at a low rate. Second, from 1995 until 2005, the decline in undernutrition was delayed, whereas the increase in overweight accelerated, leading to the coexistence of undernutrition and overnutrition (32). Third, from 2005 to 2014, the decline in stunting rates plateaued, whereas the prevalence of overweight markedly increased.

The DBM phenomenon presents an urgent challenge for achieving the Sustainable Development Goal of eradicating malnutrition in every aspect by 2030 (33). In China, the daily dietary micronutrient intake of boys and girls is generally insufficient, with many failing to meet the Chinese Estimated Average Requirement (EAR) for several essential micronutrients. Research in other countries has indicated that populations with higher BMI often have lower vitamin and mineral intake (34), suggesting a negative correlation between BMI and micronutrient intake and that obesity is associated with chronically inadequate micronutrient levels. The information that the double burden of malnutrition (DBM) is widespread in low - and middle-income countries, severely threatening the health of millions of children. According to relevant research, nearly half of the deaths among children under 5 years old are caused by malnutrition (35). For children, both undernutrition and overnutrition pose serious threats. The adverse consequences of malnutrition are extensive and far-reaching, covering physiological, psychological, and socioeconomic dimensions. These consequences may not only persist into adulthood but can even be irreversible (36). Overnutrition also has various impacts on cardiometabolic status, leading to the occurrence of chronic non-communicable diseases (21). Data from the China Health and Nutrition Survey in 2015 showed that among the 1,555 children and adolescents included in the study, the prevalence of overweight/obesity was 15.43%, and the incidence of DBM within individuals was 26.24% (37). The age range of 4 to 6 years is a critical period for children’s growth and development. The nutritional status during this stage has a profound impact on children’s cognitive function development, immune system maturation, and the risk of developing chronic diseases in adulthood. If effective intervention measures are not taken in a timely manner, DBM will not only significantly restrict the growth and future development of individual children but also further the socioeconomic burden. The occurrence and development of DBM in children are influenced by a combination of multiple factors, including genetic susceptibility, behavioral habits, dietary patterns, and environmental exposures. In this study, anemia, stunting, and overweight/obesity were used as indicators for determining DBM, aiming to deeply explore the key influencing factors of DBM in preschool children in Bengbu City and provide a scientific basis for formulating targeted intervention strategies. The present study revealed that, in addition to carbohydrates, the intake of other nutrients gradually increased with age. This pattern may reflect the increased energy requirements for growth and development, which necessitate higher nutrient intake to support ongoing physiological processes. However, no similar correlations were found between weight conditions and the intake of dietary micronutrients. In the current investigation, the intake of most micronutrients exhibited an upward trend during the study period (38). Differences in age, study groups, research design, and dietary patterns could account for these discrepancies. Dietary calcium, which is crucial for maintaining bone health (39) and supporting the maturity and growth of children, is notably deficient in the diet of Chinese adults (40, 41), which was also noted in another study (42). Plant-based proteins (e.g., legumes, grains) are rich in phytic acid, which can reduce bioavailability by chelating minerals such as zinc and calcium to form insoluble complexes (43). However, this does not mean that plant proteins cannot contribute to mineral absorption. Existing studies have shown that certain amino acids in plant proteins (such as histidine and cysteine) can significantly enhance the intestinal absorption efficiency of zinc by forming soluble complexes (44). On the other hand, animal proteins (e.g., meat, dairy products) provide ligands that promote mineral absorption, and casein phosphopeptides in dairy products inhibit the formation of calcium-phytate complexes, which increases calcium absorption by 20–30% (45). Notably, in the present study, Chinese boys and girls aged 2–5 years did not meet the Estimated Average Requirement (EAR) for dietary calcium intake, irrespective of their nutritional status. This finding emphasizes the necessity for actions focused on enhancing calcium intake, such as promoting the increased consumption of milk and dairy items. Additionally, the present study revealed a gradual increase in dietary zinc intake with age, with a particularly high prevalence in the 5-year age group. Zinc deficiency in the diet has prolonged adverse impacts on growth, immune function, and metabolic health in school-aged children (46, 47). Addressing zinc deficiency and improving dietary practices by providing guidance on dietary diversification and management are essential for optimizing children’s health and development.

Regardless of their weight status, 6.5% of the participants in this study were anemic, suggesting that individuals with low body weight may also exhibit micronutrient deficiencies due to inadequate access to nutritious, secure, cost-effective, and environmentally sustainable diets. Additionally, a comparatively small proportion of Chinese adults adhere to the recommended dietary guidelines for optimal nutritional status (48, 49). In the context of diet and disease, multiple micronutrient deficiencies are more prevalent among individuals with overweight and obesity. This prevalence could be connected to the widespread occurrence of chronic diseases in these populations, indicating the coexistence of various forms of malnutrition. Maintaining adequate dietary micronutrient intake is crucial for controlling or managing body weight, as many micronutrients, like calcium, iron, and zinc, play pivotal roles in essential metabolic and endocrine processes (50). The impact of malnutrition on the development of overt noncommunicable diseases is influenced by the subsequent nutritional status. Therefore, practical strategies should be developed to tackle various types of malnutrition, as described in this study. Regarding the differences in nutritional requirements and physiological characteristics between girls and boys, from a physiological perspective, girls may enter the growth acceleration period earlier in the late preschool stage, and their demand for trace elements such as iron and calcium is higher than that of boys. This may lead to different risk patterns of anemia and stunting between girls and boys. Boys usually have a larger amount of physical activity and higher energy consumption, and the influencing factors of their overweight/obesity may be more related to calorie intake and exercise levels.

The advantages of this study include the utilization of the most current 2023 data from a large cohort in Bengbu, which enhances the representativeness of the findings. The dietary and measurements were obtained by trained and certified interviewers, ensuring that the estimates of nutrient intake and physical measurements are relatively accurate and reliable. This study provides an understanding of the dietary micronutrient status among Chinese children, providing valuable insights for formulating targeted recommendations, interventions, and public health strategies. However, the study has some limitations. The 24-h dietary recall method was employed for three consecutive days, though providing reasonably accurate estimates of dietary intake, may not fully capture the variability of occasional food consumption. Additionally, the collected dietary data might not account for seasonal differences in micronutrient consumption. Self-reported dietary recalls are also subject to memory bias, which might influence the precision of the data. Furthermore, although the use of dietary supplements is considerably lower in China than in some Western countries, limited information on supplement and medication use among the malnourished populations in China could introduce minor biases in the assessment of dietary micronutrient intake.

Given the cohort design of our study, through long-term prospective follow-up in this study, after controlling for confounding factors such as age, gender, and family economic status, a significant association between protein intake status and the double burden of malnutrition has been found. Moreover, the risk has been quantitatively evaluated, providing relatively strong evidence for the potential causal relationship between the two. Dietary behavior is often shaped by an individual’s perception of their weight status. Nevertheless, these results offer valuable insights into dietary micronutrient intake and various states of DBM in Chinese boys and girls aged 2–5 years.

Previous research on the coexistence of malnutrition and nutrition transition in China often lacks updates, has shorter research cycles, and may not be nationally representative (17, 18, 51). Protein, an essential macronutrient for growth, has not been extensively studied with DBM, particularly regarding its impact on growth and development. Most previous research has focused on the effects of protein consumption during early life on undernourished or overnourished populations, without simultaneously considering both aspects.

Limitations: this study was a cohort study, and health questionnaires, physical examinations and bone density tests were comprehensively carried out in kindergartens in Bengbu City, all the data included in the study were complete and accurate, fully analysing the connection between the factors influencing the double burden of malnutrition in preschool children and protein intake, and providing a solid theoretical basis for the prevention of malnutrition in children and a rational diet. However, in this study, the preschool children’s health questionnaire was distributed to the children’s caregivers to fill in the questionnaire, and there was some recall bias, and when entering the questionnaire information, it was found that there were some missing data on water, salt, and spices, so such indicators could not be included in the analysis, which means that the influence of dietary factors on bone density of preschool children in Bengbu City needs to be thoroughly demonstrated, and also the children’s bone density level of the intra-school factors need to be further explored. There is a certain degree of collinearity among some variables in the study, which may lead to an increase in the variance of the estimated regression coefficients and reduce the accuracy of the parameter estimation. During the process of writing the paper, although we presented the stratified results for boys and girls, due to the limitations of the overall research framework and the length of the paper, we were unable to conduct a more in-depth and comprehensive analysis of the gender differences. From the perspective of the research design, this study focused on children aged 4 to 6 years old in Bengbu City, with an emphasis on exploring the relevant influencing factors and patterns of the overall population. As a result, the energy and space allocated to the refined study of gender differences were limited, and we failed to fully explore the potential impacts of the differences between boys and girls in aspects such as the rhythm of physiological development, characteristics of nutritional metabolism, and social behavior patterns on the research results. This is a limitation in our research planning. Besides, physical activity not only directly affects children’s energy metabolism and nutritional needs but also may be associated with dietary intake. To explicitly address two critical factors: physical activity—citing recent evidence showing that low physical activity in preschoolers correlates with reduced protein utilization efficiency (52); and infection-related confounding—adding a discussion on how systemic inflammation during infections may transiently elevate urinary nitrogen excretion, leading to overestimation of protein requirements (53). The seasonal factor is one of the limitations of this study. The seasonal changes in dietary patterns may lead to fluctuations in nutrient intake, which in turn affect the risk assessment of DBM. Future studies will adopt a longitudinal tracking design and combine seasonal dietary records to more accurately quantify the impact of seasonality on DBM. In addition, we did not explore the distinction between “inadequate intake” and “clinical deficiency” in order to avoid over-inferring the conclusions. “Inadequate intake” refers to the situation where the amount of nutrients consumed through diet fails to reach the recommended level, while “clinical deficiency” indicates the presence of observable symptoms or pathological changes due to long-term severe nutrient insufficiency. If this distinction is not clarified, it may lead to unwarranted assumptions about the health impacts of dietary patterns, as not all cases of inadequate intake will progress to clinical deficiency. By emphasizing this distinction, we can provide a more nuanced and accurate interpretation of the research findings and prevent misinterpretation of the data. However, as an observational study, even though we have tried our best to control known confounding factors, the impacts of residual confounding factors and unmeasured factors still cannot be completely ruled out. In the future, research methods such as randomized controlled trials need to be combined to further verify the causal relationship between the two.

## 5 Conclusion

This research revealed a noteworthy association between protein index quartiles and the rate of DBM in preschool children aged 2–5 years. The Chinese boys and girls in different protein index quartiles exhibited poorer diet quality and lower intake of essential micronutrients, such as calcium, zinc, and iron, relative to the EAR. Addressing and mitigating micronutrient deficiencies among Chinese children are crucial for public health. Furthermore, integrating various anthropometric indicators into the protein index can improve the accuracy of DBM predictions in clinical settings. This approach facilitates the early prevention of DBM and may reduce mortality associated with chronic diseases. Therefore, promoting the higher intake of nutrient-dense foods across the population by encouraging diverse food selection and enhancing targeted dietary supplementation programs or interventions to address micronutrient deficiencies in specific subgroups in China are imperative.

### 5.1 Future research directions

Although this study adopted a cohort design, the sample coverage was limited. In the future, a multi-center, large-sample longitudinal cohort study can be conducted to track the growth and development indicators (such as height, weight, BMI-Z score, and body composition analysis) and nutrient intake data (including serum levels of micronutrients and macronutrient energy ratios) of preschool children aged 3–5 years (covering the critical growth window period). Through long-term follow-up, the temporal causal relationship between nutritional exposure (such as protein index quartiles and micronutrient deficiencies) and the occurrence of DBM will be clarified.

## Data Availability

The original contributions presented in the study are included in the article/supplementary material, further inquiries can be directed to the corresponding author.
